# The Influence of Nutritional Factors on Immunological Outcomes

**DOI:** 10.3389/fimmu.2021.665968

**Published:** 2021-05-31

**Authors:** Evanthia Tourkochristou, Christos Triantos, Athanasia Mouzaki

**Affiliations:** ^1^Division of Hematology, Department of Internal Medicine, Medical School, University of Patras, Patras, Greece; ^2^Division of Gastroenterology, Department of Internal Medicine, Medical School, University of Patras, Patras, Greece

**Keywords:** micronutrients, macronutrients, microbiota, GALT, APC, lymphocytes, cytokines, antibodies

## Abstract

Through food intake, humans obtain a variety of nutrients that are essential for growth, cellular function, tissue development, energy, and immune defense. A special interaction between nutrients and gut-associated lymphoid tissue occurs in the intestinal tract. Enterocytes of the intestinal barrier act as sensors for antigens from nutrients and the intestinal microbiota, which they deliver to the underlying immune system of the lamina propria, triggering an immune response. Studies investigating the mechanism of influence of nutrition on immunological outcomes have highlighted an important role of macronutrients (proteins, carbohydrates, fatty acids) and micronutrients (vitamins, minerals, phytochemicals, antioxidants, probiotics) in modulating immune homeostasis. Nutrients exert their role in innate immunity and inflammation by regulating the expression of TLRs, pro- and anti-inflammatory cytokines, thus interfering with immune cell crosstalk and signaling. Chemical substrates derived from nutrient metabolism may act as cofactors or blockers of enzymatic activity, influencing molecular pathways and chemical reactions associated with microbial killing, inflammation, and oxidative stress. Immune cell function appears to be influenced by certain nutrients that form parts of the cell membrane structure and are involved in energy production and prevention of cytotoxicity. Nutrients also contribute to the initiation and regulation of adaptive immune responses by modulating B and T lymphocyte differentiation, proliferation and activation, and antibody production. The purpose of this review is to present the available data from the field of nutritional immunology to elucidate the complex and dynamic relationship between nutrients and the immune system, the delineation of which will lead to optimized nutritional regimens for disease prevention and patient care.

## Introduction

Nutrition is critical to maintaining the health and vitality of all living organisms. Nutrients ingested in the diet are essential for growth, cellular function and tissue development, energy supply, and immune defense (1). The diet of omnivores, including humans, consists of animal and plant products; these are divided into macronutrients (proteins, carbohydrates, fatty acids), micronutrients (vitamins, minerals, phytochemicals, antioxidants, probiotics), and dietary fiber, all of which have important biological functions (2).

The main goal of nutritional immunology is to study in detail the effects of nutrients on the immune system (3). An unhealthy diet or malnutrition characterized by macro- and micronutrient deficiencies can lead to ineffective immune responses and leave the organism unprotected from pathogens. In addition, many diseases are associated with a loss of essential nutrients, leading to nutrient deficiencies (4). Nutrients per se can mediate pro- and anti-inflammatory responses and modulate chronic inflammatory and autoimmune diseases (2, 3).

A field called nutrigenetics studies nutrition as a target for preventing and reversing disease progression. Nutrigenetics aims to develop personalized dietary patterns, taking into account that genetic predisposition characterizes some types of chronic diseases and that gene expression is directly influenced by environmental factors, including food metabolites (5).

## The Interaction of Nutrients and Gut-Associated Lymphoid Tissue

**Figure 1:**
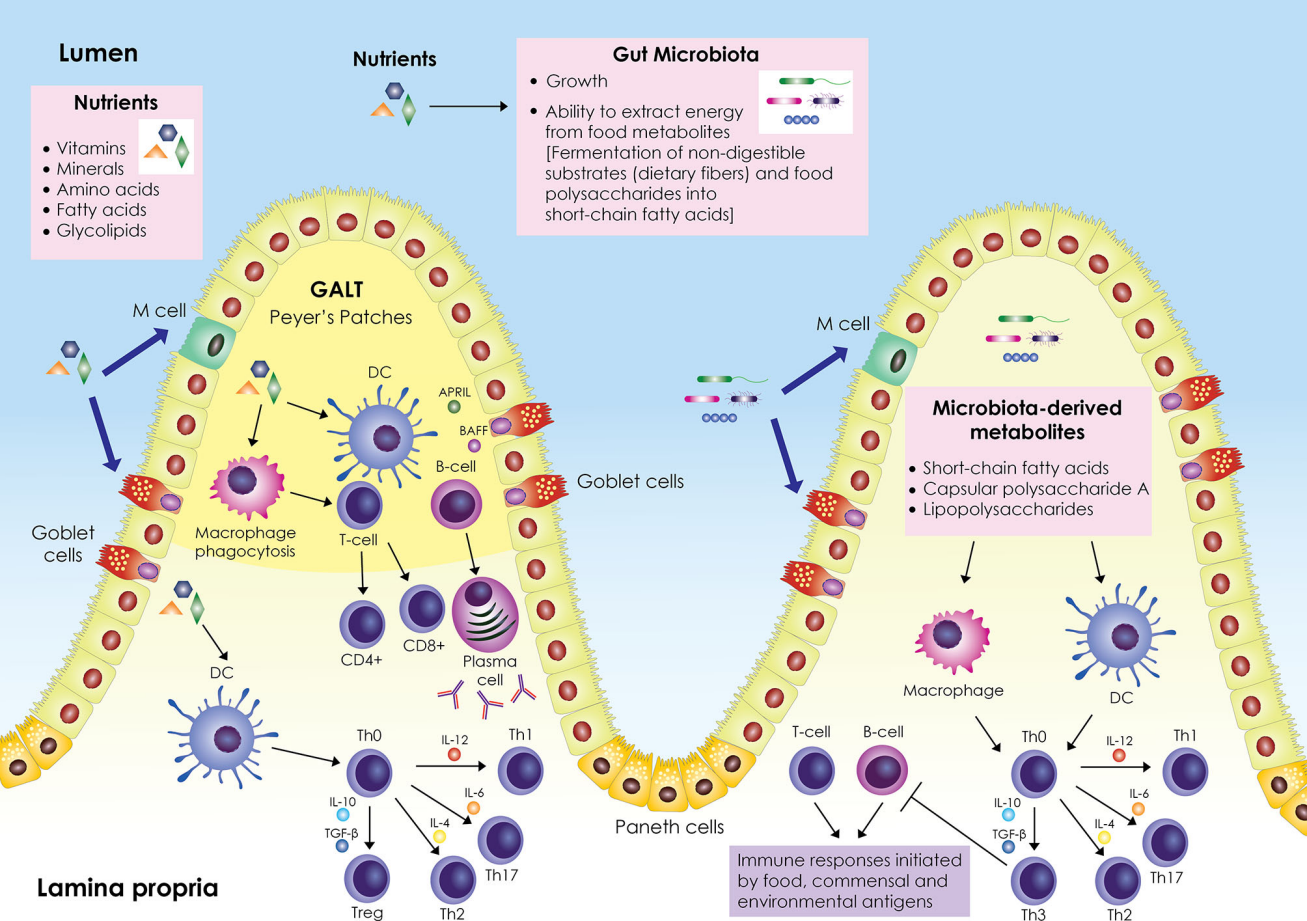
Schematic representation of the interplay between nutrients, gut microbiota and immune system. The gut-associated lymphoid tissue (GALT) occupies a large area of the gut; it is scattered within the intestinal epithelium and is also organized into lymphoid follicles in the lamina propria called Peyer’s patches. GALT consists mainly of B and T cells, macrophages, and dendritic cells (DCs). Enterocytes (Paneth cells, goblet cells, microfold (M) cells) are responsible for the active transport or passive diffusion of antigens from food during digestion and microbial components. M cells, located in Peyer’s patches, take up luminal antigens by transcytosis and present them to underlying DCs in the lamina propria, which in turn interact with B and T cells either in Peyer’s patches or in mesenteric lymph nodes. DCs secrete cytokines and induce differentiation of T helper cell precursors (Th0) into effector Th cells (Th1, Th2, Th17) or Tregs. Antigen impingement on intestinal epithelial cells and subsequent activation of DCs induces differentiation of B cells into IgA-secreting plasma cells. In parallel, nutrients modulate the gut microbiota by promoting or inhibiting its growth and affecting its ability to derive energy from dietary chemicals. Microbiota-derived metabolites (short-chain fatty acids, capsular polysaccharide A, lipopolysaccharides) stimulate and modulate DCs and macrophages to create an antigen presentation environment that favors the differentiation of Th0 cells into Th3 Tregs that inhibit T- and B-cell inflammatory responses triggered by food, commensal, and environmental antigens. Adapted from refs (6–16).

A schematic representation of the interplay between nutrients, gut microbiota, and immune system is shown in **Figure 1**. The gastrointestinal tract represents a major component of the immune system; it contains its own lymphoid tissue called gut-associated lymphoid tissue (GALT), which protects the gastrointestinal tract from invading pathogens. GALT is found in an extensive area of the intestine, organized in lymphoid follicles in the lamina propria known as Peyer’s patches (PPs), and scattered within the intestinal epithelium and in the lamina propria below the intestinal epithelium as B cells, T cells, dendritic cells (DCs), and macrophages. GALT forms the center of mucosal immunity in the intestine, where absorption of nutrients occurs and antigens from nutrients and the intestinal microbiota (bacteria, archaea, viruses, fungi, and protozoa) can elicit an immune response. The intestinal epithelial barrier, composed mainly of enterocytes (Paneth cells, goblet cells, microfold (M) cells), is a crucial tissue structure that prevents pathogen invasion (6).

Enterocytes are responsible for the active transport or passive diffusion of antigens from food during digestion and microbial components. Goblet cells secrete mucus that forms the epithelial barrier against pathogen invasion and have been found to present antigens to CD103+ DCs in the lamina propria (7). Intestinal DCs represent a crucial regulatory cell population of intestinal immunity; depending on environmental stimuli, they secrete cytokines and cell mediators, mainly IL-10, TGF-β, IL-6, IL-4, IL-12, B-cell activating factor (BAFF) and a proliferation-inducing ligand (APRIL), and induce the differentiation of T and B cells. IL-10, TGF-β, IL-6, IL-4, IL-12, induce the differentiation of T helper (Th) cell precursors (Th0) into effector Th cells (mainly Th1, Th2, Th17) or regulatory Th cells (Tregs, mainly Th3) (8–10), while BAFF and APRIL induce the differentiation of B cells into IgA-producing plasma cells in a T cell-independent manner (11). Enteroendocrine cells scattered in the intestinal tract play an important role in controlling nutrient uptake by recognizing nutrient-derived antigens in the lumen *via* microvilli structures and G protein-coupled receptors (17). M cells, located in the PPs, take up lumen antigens by transcytosis and present them to the underlying DCs in the lamina propria, which in turn can interact with B and T cells either in the PPs or the mesenteric lymph nodes (8, 12).

Nutrients exert an important role in gut immunity through their effect on microorganisms residing in the gastrointestinal tract. The gut microbiota ferments non-digestible substrates such as dietary fiber or metabolizes dietary polysaccharides into short-chain fatty acids for energy (18). In parallel, nutrients modulate the gut microbiota by promoting or inhibiting its growth and influencing its ability to obtain energy from dietary chemicals (13).

The gut microbiota shapes the development of specialized immune responses through molecular signaling that supports immune cell proliferation and communication (14, 15). Specifically, microbiota signaling regulates the expression of cytokines and antiviral mediators and mediates DNA modifications in immune cells, mainly macrophages, effector Th cells, and Tregs. Microbiota-derived metabolites and their components (short-chain fatty acids, capsular polysaccharide A, lipopolysaccharides) can stimulate and modulate antigen-presenting cells (APCs), mainly DCs and macrophages, to create an antigen presentation environment (composed mainly of cytokines IL-10, TGF-β) that favors the differentiation of Th0 cells into Th3 Tregs, which inhibit the inflammatory responses of T and B cells initiated by food, commensal and environmental antigens (14–16).

Diets enriched in saturated fatty acids, proteins, and carbohydrates have been associated with increased colonization of certain microbes at the expense of other species (19); it appears that changes in the composition of the microbiota due to a high-fat diet are associated with cardiometabolic disease (19). Studies on the effects of different dietary patterns on gut microbiota composition, short-chain fatty acid content, and inflammatory markers in healthy subjects compared to subjects with chronic diseases or metabolic syndrome showed that certain dietary components, mainly fiber and different types of fat, alter gut microbiota composition and inflammatory marker content such as c-reactive protein and IL-6 in the latter (20); therefore, modulation of microbiota composition by specific diet could lead to personalized therapeutic interventions in the future. Passive transfer of specific microorganisms, such as lactic acid-producing bacteria, Candida and Penicillium fungi, and plant viruses, into the gastrointestinal tract through diet has been previously reported (21).

## Methodology

A comprehensive literature search was conducted by electronically searching the PubMed database in all domains and using the following search strategies, including the keywords: (1) (micronutrients) OR (macronutrients) OR (nutrients) AND (immune system) OR (immune function) OR (immunity) OR (immune responses), (2) (any micronutrient/macronutrient) AND (immune system) OR (immune function), including all article types, and (3) (any micronutrient/macronutrient) AND (immune system) OR (immune function), including clinical trial and randomized controlled trial (species: Human) to examine articles written in English and published between January 1990 and March 2021 (all article types) and between January 2016 and March 2021 (clinical trials and randomized controlled trials). Critical appraisal of the collected articles depended on the original studies, including animal and human data.

## The Role of Micronutrients in Immune System Function *in Vitro* and *in Vivo*

A variety of studies, including *in vitro* culture systems of immune cells and animal models, have been performed to evaluate the immunological effects of specific micronutrients in host defense and immune homeostasis. The precise mechanisms of micronutrient involvement in the coordination of complex immune responses are still under investigation, which will provide pertinent information on their clinical role in pathological conditions. The effects of micronutrients on the immune system are summarized in **Table 1**.

**Table 1:** Effects of micronutrients on the immune system.

Nutrient	Innate immunity	Adaptive immunity	Immunological outcome	Impact on health and disease	Refs.
**Vitamin A**	Downregulation of expression and signaling of TLRs. Downregulation of TNF-α and IL-6 secretion by macrophages. ↑NK cells, ROS production by monocytes. Induction of the mTOR pathway → enhancement of cytotoxic activity and release of extracellular traps of neutrophils. Regulation of immune-related genes (TLRs, HSP70/HSP90, TRX, PGRPs).	ATRA-RAR signaling affects T and B cell development, proliferation and differentiation, Ig class switching. Enhancement of T cell migration to inflamed tissues. Modulation of B cell differentiation, secretion and synthesis of IgA and IgE antibodies. Inhibition of IL-6-driven generation of proinflammatory Th17 cells. Induction of differentiation of Th0 cells into Tregs. Induction of differentiation of immature DCs into tolerogenic DCs. Increased number of Th cells. Enhancement of humoral immunity, ↑IgM, IgG, IgA.	Universal stimulator of immunity. Immunomodulation. Maintenance of robust immune functions. Immune tolerance.	Protection against infections. Amelioration of autoimmune/inflammatory disease symptoms.	(22, 33, 90–92, 123, 124)
**Vitamin B2 (riboflavin)**	↓TLR4 and TNF-α. ↑IL-10 and antibacterial/antiviral NO. Enhancement of macrophage phagocytic activity, ↓IFN-γ, IL-6 and IL-1β pro-inflammatory responses, balanced intracellular redox state. Downregulation of the NF-κB signaling pathway.	B2 is implicated in metabolic pathways, involved in activation, differentiation and proliferation of T cells.	Antioxidant and anti-inflammatory activity.	Decreased serum levels of inflammatory markers in Crohn’s disease. Attenuation of insulin resistance and development of metabolic syndrome.	(34–38, 93, 123, 125)
**Vitamin B12**	Deficiency associated with ↑TNF-α secretion by macrophages and ↓IL-6 levels.	Deficiency associated with ↓lymphocyte number, altered ratio of Th/Tc cells and suppressed NK activity.	Immunomodulation.	Control of inflammation (↓IL-8, TNF-α, ↑TGF-β) in Alzheimer disease. ↓Severity of COVID-19.	(94, 101, 123, 126–130)
**Vitamin C**	Blockade of NF-κB activation. Enhancement of neutrophil migration and ROS production. ↑Proliferation of NK cells. Effect on cytokine production (↑IL-10, ↓TNF-α, IFN-γ). ↓Histamine levels.	Promotion of B and T cell proliferation and differentiation. ↑Antibody production.	Antioxidant and anti-inflammatory activity. Immunomodulation. Boost of innate immunity.	Maintenance of tissue epithelial barrier integrity, wound healing. Decreased severity and duration of the common cold. Cancer immunotherapy.	(39–49, 123)
**Vitamin D**	↑Chemotaxis, phagocytosis, expression of antimicrobial peptides by macrophages and monocytes. ↓Pro-inflammatory cytokines, ↑anti-inflammatory cytokines. Downregulation of TLR2,4,9 and ↓IL-6 expression in monocytes. ↑In vitro differentiation of DCs to tolerogenic DCs.	Downregulation of IL-2, IFN-γ, IL-6, TNF-α and IL-17 expression in Th cells. ↑Th1 ↓Th2 responses ↑B and T cell differentiation/activation/proliferation. Induction of differentiation of naive T cells into Tregs.	Immunomodulation. Immune tolerance.	Protection from acute respiratory infections. ↓Severity of COVID-19. ↓Anti-Tg antibodies in Hashimoto’s thyroiditis. Anti-inflammatory activity (↓TNF-α, IFN-γ, IL12p70) in ulcerative colitis. Decrease in markers of T cell activation/exhaustion and monocyte activation in HIV infection. Immunoregulation in type 1 diabetes.	(56–64, 101–110)
**Vitamin E**	↓Migration of DCs, ↓IL-12 production, ↑activity of NK cells. ↓Inflammatory mediators (leukotrienes).	Enhancement of immunological synapse APC-T cell, T cell activation. Polarization of Th cells to Th1 and Th2.	Regulation of cellular immunity. Modulation of membrane integrity, signal transduction, and oxidative stress in immune cells.	Protection against infection. Improved steatosis and inflammation in patients with nonalcoholic steatohepatitis and type 2 diabetes.	(65, 69, 111, 112)
**Folate (vitamin B9)**	↓NK cell number and cytotoxicity. ↑IL-8, TNF-α.	Maintenance of Treg numbers in the colon.	Affects DNA synthesis and cell cycle, DNA methylation and regulation of gene expression → proper immune cell function.	Immune system homeostasis. ↓Inflammatory cytokines in mild cognitive impairment. Viral-mediated autoimmunity in type 1 diabetes.	(70, 71, 95, 96, 123)
**Zinc**	Inhibition of NF-κB→↓TLR4 signaling, IL-1β, TNF-α, IL-6 production. Enhancement of NK cell development. ↑IFN-γ-producing NK cells.	Zinc deficiency → thymic atrophy, decreased number and activity of lymphocytes, oxidative stress. Zinc deficiency → ↑Th17 cells, ↓Treg function.	Enhancement of innate immunity. Development of T cells.	Reduction of inflammation in HIV. High probability of survival in COVID-19. Improvement in response to Hepatitis B vaccination. Improvement in anti-cancer activity of monocytes in type 2 diabetes. ↓IL-1, TNF-α in polycystic ovary syndrome.	(78–80, 114–118, 123, 131, 132)
**Selenium**	Regulation of synthesis of inflammatory mediators. ↑Phagocytic activity.	↑Th1 responses. Enhancement of differentiation of Th cells, T cell activation. Selenium deficiency decreases B cell numbers.	Enhancement of innate immunity. Upregulation of cellular immunity.	Increase in antibody titers, improved vaccine effects. ↓IL-1, TNF-α in polycystic ovary syndrome. ↑Neutrophils, IgG, IgA in cancer. High probability of survival in COVID-19.	(72, 74, 75, 119–123, 133, 134)
**Copper**	Deficiency associated with ↓neutrophil numbers and antimicrobial function.	Deficiency associated with ↓T cell proliferation and ↓IL-2 secretion.	Enhancement of innate and adaptive immunity.	Energy production and prevention of oxidative stress in cells.	(76, 77, 135)
**Iron**	Iron homeostasis affects signaling and metabolic pathways relative to DNA synthesis and microbial killing. Deficiency associated with ↓neutrophil numbers, phagocytic activity, and IL-6 levels.	Deficiency associated with ↓IgG levels. Genes and proteins involved in iron homeostasis have been linked to the regulation of T cell differentiation and activity.	Regulation of immune cell proliferation and innate immune response.	Strengthens immunity against infections. Iron deficiency → anemia → ↓cellular, humoral, innate immunity and cytokine production.	(81, 82 123, 136–138)

### Vitamin A

Vitamin A is a dietary micronutrient that is present in the form of carotenoids and retinyl esters and is associated with the maintenance of robust immune functions. Vitamin A binds to retinoic acid receptors (RARs), which form heterodimers and act as transcription factors to regulate the expression of several genes involved in cell growth and differentiation, lipid homeostasis, and insulin responses (139). Carotenoids are universal stimulators of immunity; metabolic cleavage of β-carotene generates retinaldehyde molecules that activate the RXR receptor (140), with resultant regulation of many immune-related genes across species. Experiments with invertebrates showed that carotenoids regulate the expression of immune-related genes such as TLRs, heat shock proteins HSP70 and HSP90, thioredoxin-like protein (TRX), and peptidoglycan recognition receptor proteins (PGRPs) (22), possibly having a positive impact on the innate immune response against pathogens. Experiments with rabbits showed that carotenoids can enhance the humoral immune response by increasing serum IgM, IgG and IgA levels (23). A metabolite of vitamin A (all-trans-retinoic acid, ATRA) binds to the RAR in the nucleus of neutrophils isolated from human blood and induces the mTOR pathway, increasing the cytotoxic activity and extracellular trap release of neutrophils (24). Retinoid supplementation has also been shown to control an inflammatory response *in vitro* by downregulating TLR expression and secretion of the proinflammatory cytokines TNF-α and IL-6 by macrophages when they phagocytose pathogens (25). The effect of vitamin A on adaptive immunity depends on the expression of RARs by T and B cells, with signaling induced by ATRA binding to RAR affecting cell development, proliferation and differentiation, and Ig class switching (26). Retinoid supplementation in normal B cell cultures resulted in modulation of B lymphocyte activation, differentiation and cytokine production (27). In parallel, retinoic acid was shown to induce IgA secretion (28), suppress IgE secretion, and accelerate allograft rejection by enhancing T cell migration into the graft *in vivo*, a finding that could be considered in studies of factors determining successful transplantation (29, 30). A role of vitamin A in immunomodulation, especially in autoimmune and inflammatory diseases, could be proposed, since vitamin A has been shown to have a tolerogenic effect on immune cells *in vitro*; in particular, it promotes the development of Th2 cells (31), inhibits the IL-6-driven generation of proinflammatory Th17 cells, and induces the differentiation of Th0 cells into Tregs (32). Moreover, vitamin A induces the differentiation of human immature DCs into tolerogenic DCs, which in turn induce the differentiation of naive B cells into B cells expressing vitamin A receptors and having immunosuppressive/regulatory activity (33).

### Vitamin B2 (Riboflavin)

Riboflavin is a micronutrient with antioxidant and anti-inflammatory activity. Vitamin B2 is converted by riboflavin kinase into two active forms, flavin adenine dinucleotide and flavin mononucleotide, which are cofactors involved in redox reactions in the tricarboxylic acid cycle and fatty acid oxidation (34). These metabolic pathways are involved in T cell activation, differentiation and proliferation (35). An anti-inflammatory role of vitamin B2 through down-regulation of the NF-κB pathway and production of proinflammatory cytokines has also been suggested, as vitamin B2 has an inhibitory effect on proteasome activity, which blocks the activity of NF-κB components in the cytoplasm (36). Culture with vitamin B2 altered the responses of murine macrophages to lipopolysaccharide (LPS) stimulation: expression of TLR4 and secretion of the proinflammatory cytokine TNF-α were reduced, while expression of the anti-inflammatory cytokine IL-10 and production of the antibacterial/antiviral agent nitric oxide (NO) were increased (37). B2 supplementation in mice increased the phagocytic activity of Staphylococcus aureus-infected macrophages and decreased the levels of the cytokines IFN-γ, IL-6, and IL-1β, possibly by regulating the NF-κB pathway (36). A possible role of B2 supplementation in attenuating insulin resistance and the development of the metabolic syndrome was highlighted in an *in vitro* adipocyte-macrophage co-culture system that mimics obesity-related inflammation. Specifically, riboflavin supplementation resulted in a reduction in proinflammatory factors (TNF-α, IL-6, MCP-1, HMGB1) and an increase in anti-inflammatory factors adiponectin and IL-10 (38), highlighting the need for further evaluation of this micronutrient as a promising target in the treatment of inflammatory conditions associated with obesity and metabolic syndrome.

### Vitamin C

Vitamin C is a water-soluble micronutrient of high molecular importance as it is a cofactor of hydroxylases, enzymes involved in the regulation of gene transcription, and cell signaling pathways (39). An anti-inflammatory effect of vitamin C has been reported through its antioxidant activity in the cytoplasm, where it quenches ROS and is oxidized to dehydroascorbic acid (DHA) with the release of electrons. DHA directly inhibits the kinase IKKβ, blocking NF-κB activation and the expression of proinflammatory cytokines (40). Moreover, culture of LPS-stimulated peripheral blood mononuclear cells with vitamin C resulted in decreased secretion of TNF-α and IFN-γ and increased secretion of IL-10 by the cells (41). Enhancement of innate immunity appears to be achieved by vitamin C, as it has been shown to promote microbial killing by increasing the migration of neutrophils to sites of infection in response to chemotactic signals and the release of ROS (42). Addition of vitamin C to NK cell cultures resulted in increased NK cell proliferation (43), indicating a potent role of this micronutrient in studies testing NK cell-based immunotherapy against malignancies. High-dose administration of vitamin C in murine cancer models has also highlighted the beneficial effect of this vitamin on the inhibition of tumor growth in a T cell-dependent manner, as it appears to modulate tissue infiltration by adaptive immune cells and increase the cytotoxic activity of adoptively transferred CD8 T cells in mice (44). In addition, vitamin C has been shown to help maintain the integrity of tissue epithelial barriers and accelerate wound healing by promoting collagen synthesis, proliferation, and migration of fibroblasts (45, 46). Vitamin C has also been shown to improve adaptive immunity by promoting T and B cell differentiation and proliferation (47, 48) and increasing antibody production (49).

### Vitamin D

Vitamin D is an important micronutrient for bone and mineral metabolism by promoting intestinal absorption of calcium and phosphate and stimulating osteoclast differentiation and calcium absorption from bone, thus maintaining calcium balance in the skeleton (50). Vitamin D exerts its role by binding to its receptor (VDR), which is expressed in many cell types including B and T cells (51). In activated and proliferating B and T cells, the expression of VDR is increased (52, 53), indicating an important involvement of vitamin D in adaptive immune responses. The vitamin D-VDR complex forms heterodimers with the retinoid X receptor (RXR), a nuclear receptor that forms heterodimers with other steroid hormone receptors that act as transcription factors and regulate gene expression (54). The vitamin D-VDR-RXR complexes enter the nucleus, bind to the vitamin D response element and regulate the expression of vitamin D-responsive genes (55). An immunomodulatory effect of vitamin D on Th cell responses has been highlighted *in vitro*; vitamin D has been shown to exert a potent anti-inflammatory effect by down-regulating Th1-mediated immune responses and inhibiting the expression of the proinflammatory cytokines IL-2, IFN-γ, IL-6, TNF-α and IL-17 in Th cells (56, 57), and by enhancing Th2 activity by promoting the production of the cytokines IL-4, IL-5 and IL-10 (58). A decrease in Th17 cell number and an increase in Treg cell number have also been noted (59, 60). In terms of innate immunity, vitamin D has been shown to inhibit the expression of IL-6 and TLRs 2, 4 and 9 in monocytes, resulting in hyporesponsiveness to pathogen-associated molecular patterns, a fact that could have a negative impact on effectiveness in encountering pathogens, or a positive impact on acute pathological conditions, including sepsis and autoimmunity, in which TLR activity is increased (61). Vitamin D has also been shown to increase chemotaxis and phagocytosis as well as the expression of antimicrobial peptides in cultured monocytes and myeloid cells (56, 62, 63). An important activity of vitamin D in immune tolerance has also been highlighted, as it is one of the biological factors (others are IL-10, TGF-β, apoptotic cells) that induce differentiation of human immature DCs into tolerogenic DCs *in vitro*, which in turn induce differentiation of naive T cells into Tregs (64).

### Vitamin E and Vitamin B9

Vitamin E is a fat-soluble compound found in the cell membrane where it scavenges peroxyl radicals, preventing oxidation of polyunsaturated fatty acids and oxidative damage to the cell. A balanced redox state in the cell can influence signal transduction, as the activity of signaling enzymes is regulated by free radicals (65). Thus, vitamin E exerts an important role in modulating membrane integrity, signal transduction, and oxidative stress in immune cells, as their cell membranes are enriched in vitamin E isoforms (α- and γ-tocopherol) (65). Experiments using cell-free and cell-based systems have shown significant anti-inflammatory activity of endogenous metabolites of vitamin E targeting target 5-lipoxygenase, a key enzyme in the biosynthesis of inflammatory lipid mediators, including chemoattractant and vasoactive leukotrienes (66). Vitamin E has been associated with the regulation of cellular immunity and protection against infection (67), it increases the interaction of APCs with T cells with subsequent T cell activation, and it can alter the function of DCs by influencing their migration and decreasing IL-12 production; it also increases the activity of NK cells (67) and acts as a modulator of Th0 cell differentiation into Th1 or Th2 cells (68, 69).

Folic acid (vitamin B9) is a micronutrient that affects DNA synthesis and cell cycle, DNA methylation, and regulation of gene expression. Substrates from the metabolism of B9 can interfere with enzymatic reactions in these processes and act as co-factors (70). Vitamin B9 also contributes to the maintenance of Tregs in the colon; mice fed a folic acid-deficient diet were more prone to intestinal inflammation (71).

### Selenium, Copper, Zinc, Iron and Folic Acid Trace Elements

Trace elements are micronutrients that are present in small amounts in the living organism, performing important biological functions as they participate in chemical and molecular processes in the form of cofactors of many enzymes and antioxidant molecules.

Selenium in the form of the amino acid selenocysteine forms part of the catalytic active site of peroxidases that catalyze oxygen-reactive species and may regulate metabolic reactions that induce the synthesis of lipoxygenases, enzymes involved in the synthesis of inflammatory mediators (72, 73). Selenium upregulates cellular immunity by enhancing TCR-induced activation of T cells and Th0 differentiation into Th1 cells in mice (74) and is involved in redox signaling, an important mechanism for the killing of microorganisms by phagocytes (72, 75).

Copper plays a major role in chemical reactions important for energy production and prevention of oxidative stress in the cell, since enzymes (cytochrome c oxidase, copper-zinc superoxide dismutase) involved in electron transfer during the mitochondrial transport chain and in catalytic free radical reactions are copper-containing (76). An interesting role of the copper trace element in the regulation of gene transcription has also been highlighted in yeast species, in which the level of gene expression changed in response to copper availability (77).

Zinc is involved in TLR4 signaling by inhibiting the activation of the transcription factor NF-κB (78) and decreasing the production of the proinflammatory cytokines IL-1β, TNF-α, and IL-6 (79). In vitro, zinc mediates the differentiation of CD34+ progenitor cells into NK cells (32), and *in vivo*, zinc supplementation results in increased numbers of IFN-γ-producing NK cells (80).

Iron can affect cell cycle and proliferation by acting as a co-factor in a variety of enzymes involved in DNA synthesis and repair (81). Iron is necessary for the regulation of immune cell proliferation and innate immune response. Transferrin receptors responsible for iron uptake are expressed in immune cells (monocytes, macrophages, T cells) (82). Iron is also an important substrate for pathogen growth, and a variety of genes and proteins involved in iron homeostasis have been linked to immunomodulatory effects, including TLR signaling, regulation of T-cell differentiation and activity, and resistance to infection (82).

## Effects of Micronutrient Supplementation on Immunity and Disease: Clinical Data

The supply of necessary micronutrients is crucial for the normal development of the immune system during the different stages of human growth (gestation, neonatal maturation, weaning). Inadequate maternal nutrition and micronutrient deficiencies, especially vitamins A, D, E, B2, B6, B12, and folic acid, are associated with an increased risk of infant morbidity due to respiratory infections, intestinal inflammation, allergies, asthma, and neurodevelopmental disorders (83–87). Prenatal IgE antibody formation is influenced by food metabolites and predicts the development of food allergies in infancy (88). Diet continues to influence immune system function during aging. Diet and nutritional status can influence immunosenescence, a gradual deterioration of the immune system with age that results from continuous antigenic challenges, infections, and chronic inflammation (89).

In a study of 36 healthy Bangladeshi men with low vitamin A stores vitamin A supplementation resulted in increased numbers of NK cells, production of reactive oxygen species (ROS) by monocytes, decreased serum cytokines IL-6 and IL-17, limited inflammatory responses to pathogens mediated by Th1 and Th17 cells, and increased levels of IFN-γ-inducible protein 10 (IP-10, CXCL10); the latter recruits activated T cells to sites of tissue inflammation (90, 91). A clinical study of 306 Bangladeshi infants examined the effects of vitamin A administration on Treg development, mucosal targeting of immune cells in response to chemokine receptor 9 (CCR9) expression, and systemic endotoxin burden as indicated by altered plasma concentrations of soluble CD14 (sCD14), a marker of systemic bacterial endotoxin. In low birth weight infants receiving vitamin A supplementation, the expression of CCR9 on Tregs was increased, indicating an increase in their numbers in the intestinal mucosa with a consequent decrease in intestinal inflammation. The decrease in sCD14 concentration after vitamin A supplementation at 2 years of age showed an important role of vitamin A in improving intestinal integrity, also considering the better vitamin A status at the same age (92). Considering the possible positive role of vitamin A in intestinal immunity and homeostasis, it could be proposed as a therapeutic factor in patients with inflammatory bowel disease.

A beneficial role of B2 was demonstrated in a prospective clinical intervention study that included 70 patients with Crohn’s disease. B2 supplementation (oral dose 100 mg daily for 3 weeks) showed anti-inflammatory effects, decreased systemic oxidative stress, and symptom improvement, represented by decreased serum levels of inflammatory markers, increased plasma free thiols, and decreased clinical disease activity (Harvey-Bradshaw Index), respectively (93).

Together with B2, vitamin B12 has shown promising results in controlling inflammation-associated disease symptoms. Patients with Alzheimer’s disease who received antipsychotic drugs plus vitamin B12 had decreased expression of the proinflammatory cytokines IL-8 and TNF-α and increased expression of the anti-inflammatory cytokine TGF-β and exhibited milder psychotic symptoms (94). A prospective clinical trial involving 30 healthy Brazilian adults examined the effect of a daily dose (5 mg) of folic acid on immunity and showed a folate-associated significant reduction in the number and cytotoxicity of NK cells, as well as higher levels of the cytokines IL-8 and TNF-α (95). The association of folic acid with reduced NK responses has also been suggested as a potent factor in the virus-mediated pathology of autoimmune type 1 diabetes, whereby defects in the folate pathway may lead to NK dysfunction and thus enhance the negative influence of viral infections in the initiation of autoimmune responses (96).

Vitamin C also plays an important role in the prevention of infections and inflammation (97, 98). Intravenous infusion of vitamin C in patients with allergic diseases resulted in decreased serum histamine levels (99). Vitamin C supplementation of 0.2 g/day or more was associated with decreased severity and duration of the common cold (100).

A cohort observational study that included hospitalized patients with COVID-19 aged >50 years showed a beneficial effect of combining vitamin D, magnesium and vitamin B12 (DMB) on clinical outcomes of the disease. DMB supplementation resulted in a reduction in the number of patients requiring oxygen and/or intensive care support, suggesting an ameliorative role of these micronutrients on disease severity (101). Other clinical studies have also shown a protective role of vitamin D supplementation against acute respiratory infections and symptom relief in vitamin D-deficient children and immunodeficient patients (102, 103), also considering that it affects the balance of type 1/type 2 cytokines; specifically, it causes a decrease in the production of type 1 cytokines TNF-α, IFN-γ, IL-6, IL-8, IL-9, IL-12, IL-17, and an increase in the production of type 2 cytokines IL-4, IL-5, IL-10 (104, 105). A positive effect of vitamin D on the augmentation of salivary immune responses was also found during stressful military training in healthy subjects, who showed higher salivary secretory immunoglobulin A and cathelicidin secretion rates (indices of mucosal immunity) after vitamin D supplementation (106). A possible therapeutic role of vitamin D in autoimmune diseases was highlighted in a study of patients with Hashimoto’s thyroiditis, in which vitamin D supplementation resulted in a significant reduction in anti-thyroglobulin antibodies (107), and in patients with type 1 diabetes, where vitamin D supplementation was shown to lower the levels of Th17 inflammatory cells (108). An anti-inflammatory effect of vitamin D was also found in patients with ulcerative colitis, who showed a significant decrease in serum proinflammatory cytokine levels (TNF-α, IFN-γ, and IL12p70) (109). Vitamin D supplementation has also been suggested as a potent adjuvant therapy to antiretroviral drugs in HIV infection after a decrease in markers of T cell activation/exhaustion and monocyte activation was shown in HIV-infected adolescents receiving a maximum dose of 120,000 IU/month (110).

Vitamin E is another micronutrient with potent immunomodulatory activity. Supplementation of vitamin E and a tocotrienol-rich fraction in healthy subjects has been associated with regulation of gene expression and molecular signaling pathways related to immune response, response to stress, stimuli, hypoxia, bacteria, and complement. A possible antioxidant and anti-inflammatory activity of vitamin E through the downregulation of signaling pathways related to apoptosis, NF-κB kinase, the cascade of extracellular signal-regulated kinase-1 and extracellular signal-regulated kinase-2 has been indicated (111). A beneficial effect of co-administration of nutrients and therapeutic medications in diseases was suggested in a randomized controlled trial investigating the effect of vitamin E on liver histology in patients with nonalcoholic steatohepatitis (NASH) and type 2 diabetes, where the combined treatment of vitamin E and pioglitazone improved steatosis and inflammation in patients leading to resolution of NASH (112).

Supplementation of B9 in patients with mild cognitive impairment improved cognitive function, which was associated with a reduction in peripheral inflammatory cytokines (113).

Regarding the effects of trace element supplementation on immune function and disease, zinc supplementation in patients with HIV showed potency in lowering markers of systemic inflammation, monocyte activation, and microbial translocation (114). Serum zinc and selenium levels have also been reported as predictive markers of survival in COVID-19 patients, with reference ranges of these trace elements associated with high-survival probability, highlighting the need for further evaluation of their supplementation effects in the disease (115). Prenatal and postnatal zinc supplementation has been shown to improve T cell-dependent antibody responses to hepatitis B vaccination in infants (116). An anticancer role of zinc supplementation was demonstrated in type 2 diabetic patients with metabolic syndrome who received 30 mg elemental zinc/day or placebo for eight weeks and exhibited increased proportion of monocytes expressing transmembrane TNF-α (117). Co-administration of zinc and magnesium in patients with polycystic ovary syndrome significantly decreased serum high-sensitivity C-reactive protein, a marker of inflammation, and downregulated proinflammatory IL-1 and TNF-α cytokines (118). Moreover, selenium supplementation in healthy individuals improves their response to vaccination and increases their antibody titers (119, 120), indicating a potent role of micronutrients as cofactors in vaccine-mediated immunity. An anti-inflammatory effect of selenium was also demonstrated in infertile women with polycystic ovary syndrome who were candidates for *in vitro* fertilization, who showed a decrease in gene expression of the proinflammatory cytokines IL-1 and TNF-α after selenium supplementation (121). A positive influence of selenium administration on immunity in cancer was observed, as patients with lymphomas and solid tumors who received selenium showed a significant increase in neutrophils and IgG and IgA antibody titers (122).

## Micronutrient Deficiencies Can Impair Immune Function

Deficiencies in micronutrients such as vitamins A, B12, C, folic acid, riboflavin, iron, zinc, and selenium can increase immunosenescence, particularly susceptibility to infection and progression of inflammation (123).

Vitamin A deficiency has been shown to decrease the number of NK cells and impair their activity, impair the ability of neutrophils and macrophages to undergo phagocytosis, impair the growth and differentiation of B cells, decrease the number of T cells and limit their distribution (124).

Vitamin B2 deficiency has been associated with obesity-associated chronic inflammation. Experiments with mouse adipocytes showed that B2 deficiency resulted in stimulation of the proinflammatory NF-κB signaling pathway and increased levels of IL-1, TNF-α and ROS (125).

Vitamin B12 plays an immunomodulatory role in cellular immunity (126, 127) and its deficiency has been associated with upregulation of proinflammatory TNF-α in macrophages from B12-deficient mice and a decrease in IL-6 levels in B12-deficient rats, conditions that reversed in both animal models after increasing B12 supplementation (128–130). Vitamin B12-deficient anemic patients exhibit lower numbers of all lymphocytes, Tc cells, a change in Th/Tc cell ratio, and suppressed NK cell activity, all of which are reversed after B12 supplementation (126).

Deficiencies of certain trace elements have also been shown to have negatively affect the number and function of immune cells, leading to dysregulation of immune homeostasis.

Zinc deficiency leads to thymic atrophy, decreased number and activity of lymphocytes, altered cytokine production, increased oxidative stress, and inflammation (131) and is also associated with autoimmune and inflammatory diseases by affecting Th cell polarization by promoting differentiation of Th0 cells into proinflammatory Th17 cells, accompanied by loss of Treg function (132).

In mice, it has been shown that selenium deficiency leads to a decrease in B cell count (133). However, excessive selenium intake can be counterproductive, as infectious microorganisms can utilize this nutrient in their biochemical processes (134).

Copper deficiency is associated with a reduction in the number and antimicrobial function of neutrophils and also with a reduction in the IL-2 secretion of T cells with consequent reduction in T cell proliferation (135).

Abnormalities in Th and Tc cell numbers and function have been reported in patients with hereditary hemochromatosis, which is characterized by low iron stores (136). Iron deficiency is associated with weakened cellular immunity, decreased numbers of neutrophils and their phagocytic activity, lower levels of IL-6 and IgG antibodies (137, 138).

## Effects of Macronutrients on Immunity and Disease

The effects of macronutrients on the immune system are summarized in **Table 2**. Macronutrients, including proteins, carbohydrates, and fatty acids, provide tissues with the energy necessary for their development and function (180). Immunomodulation by macronutrients has been studied in experimental animals and in human intervention studies by testing the effect of their intake on immunological outcomes.

**Table 2:** Effects of macronutrients on the immune system.

Nutrient	Innate immunity	Adaptive immunity	Immunological outcome	Impact on health and disease	Refs.
**Proteins**	Regulation of NK cell activation, macrophage activation, and cytokine and cytotoxic factor production. ↑NK killing activity, macrophage phagocytosis, antioxidant activity. ↓Pro-inflammatory adipokines chemerin and progranulin.	Regulation of B and T cell activation, lymphocyte proliferation, and antibody and cytokine production.	Immunomodulation. Regulation of activation of innate and adaptive immunity.	Robust immune responses. Modulation of inflammatory immune responses in type 2 diabetes. ↓Pro-inflammatory monocytes in obese/overweight individuals.	(141–149)
**Carbohydrates**	Regulation of cell adhesion during leukocyte migration, recognition of carbohydrates in the membrane of pathogens → regulation of immunity to infection. Influencing the binding of antigen presentation proteins (MHC-I/II), modulation of NKT cell activation. ↓Phagocytic activity of monocytes and granulocytes, oxidation, limitation of pro- and anti-inflammatory cytokine response. Cell surface molecules → recognition by TLRs, activation of γδ and αβ T cells. ↓TLR-4 by monocytes.	Influencing the binding of antigen presentation proteins (MHC-I/II), modulation of T cell recognition, activation of Th, Tc and NKT cells and cytokine production.	Immune recognition. Balanced number of immune cells.	Robust immune responses. Anti-inflammatory activity in obese/overweight individuals and type 2 diabetes. Restoration of T cell subsets in HIV infection. Immunomodulation in multiple sclerosis.	(150–157)
**Fatty acids**	Regulation of APC activation signaling. Regulation of NLRP-3 inflammasome and production of pro-inflammatory IL-1β, IL-18 and activity of transcription factor NF-κB. Regulation of phagocytic activity of macrophages, leukocyte migration, infiltration of DCs into lymph nodes and activation of mast cells. Polyunsaturated fatty acids → ↓ expression of adhesion molecules by endothelial cells → leukocyte migration.	Influencing immune cell function → energy source, cell membrane components, signaling molecules/gene expression. Regulation of T and B cell activation, T cell proliferation, cytokine production, activation of apoptosis/cell death. ↑IgM production. Influence on immune cell crosstalk. Promotion of Th0 cell differentiation into Tregs, ↑Treg suppressive capacity.	Immune cell function. Immunomodulation	Immune regulation (Th0 → Tregs) in multiple sclerosis/healthy subjects. Polyunsaturated fatty acids → ↓risk of allergic diseases, ↓atherosclerosis. Maintenance of Th cells and hsCRP in breast cancer. Resolution of inflammation in chronic kidney disease.	(158–179)

### Proteins

Proteins represent important macronutrients for the immune system, considering that amino acids are essential for the synthesis of immune proteins, including cytokines and antibodies that mediate immune responses (141). Metabolic degradation of certain amino acids (tryptophan, arginine) leads to the production of chemical substrates involved in biological processes (142). The metabolism of tryptophan contributes to the synthesis of the cofactor NAD+, which is involved in redox reactions and electron transfer. The degradation of arginine has been associated with the induction of the non-canonical NF-κB pathway and the regulation of gene transcription in the context of immune tolerance (142). Metabolism of arginine and methionine also leads to the synthesis of polyamines, which are organic compounds that regulate cell proliferation through their role in maintaining DNA, mRNA, and cell membrane stability; polyamines have been linked to the induction and regulation of inflammation and pathogen recognition by affecting receptor-ligand binding. Degradation of methionine leads to the synthesis of glutathione, an antioxidant organic compound involved in the prevention of oxidative stress, regulation of NK and T cell cytotoxicity and macrophage activity, and activation of T cells (143).

Amino acids exert multiple roles in the immune system, including regulation of the activation of adaptive and innate immune cells (B and T cells, NK cells, macrophages), proliferation of lymphocytes, and production of antibodies, cytokines, and cytotoxic factors (141, 144). Hydrolysates or bioactive peptides released by gastrointestinal digestion have been reported to have antioxidant activities, increase killing activity of NK cells, increase phagocytic activity of macrophages, and increase the size of lymphocyte populations, antibody and cytokine production (145).

Studies in pigs and the fruit fly Drosophila melanogaster, showed that high dietary protein intake has a positive effect on intestinal cellular and humoral immunity, associated with increased expression of antimicrobial peptides (146). On the other hand, low protein diet has been reported to induce immunity against tumor cells in mice. Specifically, a decrease in protein content and the associated decrease in amino acids cause ER stress, which leads to the activation of T cells that secrete proinflammatory cytokines (147).

A positive effect of a high-protein diet on the modulation of inflammatory immune responses was demonstrated in an intervention study of type 2 diabetics who were fed a diet high in animal or plant protein for 6 weeks. After this period, they had reduced levels of the proinflammatory adipokines chemerin and progranulin regardless of protein source (148). Overweight and obese adults who followed an energy-restricted diet with either normal or high protein achieved weight loss accompanied by significant decreases in proinflammatory monocyte subpopulations, plasma lipids, and lipoproteins (149).

### Carbohydrates

An important aspect of the effect of macronutrients on the immune system is their involvement in immune recognition. Carbohydrates represent common cell surface molecules that can be recognized as antigens by TLRs. Glycolipids, zwitterionic polysaccharides and glycopeptides can be presented to γδ and αβ T-cells either through endosomal pathways or extracellular receptors (150). Glycoproteins and glycolipids can bind to glycan-binding proteins such as lectins and antibodies, regulating cell adhesion during leukocyte migration and immunity to infection through recognition of carbohydrates contained in the membrane of pathogens (151). In addition, carbohydrates participate in enzymatic processes of protein glycosylation that generate important functional biopolymers. Glycosylated peptides act as glycoantigens that influence the binding of antigen presentation proteins of the HLA-I and HLA-II systems and subsequent T-cell recognition of antigenic peptides. Glycoantigens processed through intracellular pathways in APCs are presented to Th, Tc and NKT cells and modulate their activation and cytokine production (152).

An anti-inflammatory role of carbohydrate intake was highlighted in a study that reported that consumption of a carbohydrate-containing beverage resulted in a decrease in the expression of TLR4 receptors in monocytes of obese/overweight children compared to normal weight children (153). Another study reported that carbohydrate intake by athletes was associated with a more balanced number of immune cells in the blood, decreased phagocytic activity of monocytes and granulocytes, and decreased ROS and inflammatory cytokine levels (154).

A low-carbohydrate, high-fat diet with or without after-meal walks in patients with type 2 diabetes improved glucose control and fasting pro-insulin levels and significantly decreased phosphorylated c-Jun N-terminal kinase in peripheral blood mononuclear cells, which is a marker of cellular inflammation (155).

A hydrolyzed polysaccharide, a rice bran arabinoxylan compound, administered as a dietary supplement in HIV patients resulted in a significant decrease in peripheral blood Tc cells and a significant increase in the Th/Tc cell ratio (156).

An anti-inflammatory effect of a polysaccharide-based diet was also observed in patients with multiple sclerosis, in whom a 12-month polysaccharide-based multinutrient diet contributed to a significant decrease in IL-2, TNF-α, EGF, and CD95 + CD34+ cell subsets (157).

### Fatty Acids

Fatty acids provide an important source of energy, are components of the cell membrane, and modulate cell function by acting as signaling molecules that can regulate gene expression (158). They can also influence immune cell functions by serving as precursors for the synthesis of lipid compounds involved in the regulation of immune responses and inflammatory pathways (158, 159). Metabolic derivatives of fatty acids, including eicosapentaenoic acid (EPA) and docosahexaenoic acid (DHA), are precursors for anti-inflammatory molecules that contribute to monocyte recruitment to sites of inflammation, where they engulf and remove apoptotic neutrophils (160). Fatty acids regulate the phagocytic activity of macrophages, infiltration of DCs into lymph nodes and activation of mast cells (161, 162). They also cross-react with the peroxisome proliferation activation receptor and TLRs (163, 164).

Fatty acids can have a dual effect on the regulation of inflammation, depending on whether or not double bonds are present between the individual carbon atoms (unsaturated and saturated fatty acids). Saturated fatty acids have been shown to stimulate *in vitro* and *in vivo* the intracellular macromolecular complex Nod-like receptor protein 3 (NLRP3) inflammasome, which promotes the production of the proinflammatory cytokines IL-1β and IL-18, whereas unsaturated fatty acids exert an inhibitory effect on the NLRP3 inflammasome by limiting the activity of the transcription factor NF-κB (165), an observation that could be taken into account in clinical nutrition studies to attenuate inflammatory conditions in diseases.

The concentration of fatty acids is a factor that may influence their effect on immune cells. Low concentrations of free fatty acids induce T cell proliferation and cytokine production, whereas high concentrations of free fatty acids cause mitochondrial membrane dysfunction, leading to activation of apoptotic pathways and cell death (166).

Fatty acids, as components of lipid rafts in the cell membrane, can modulate cell signaling, which affects immune cell function, considering that lipid rafts have been shown to contribute to Th cell activation (167). Metabolic derivatives of omega-3 fatty acids have also been shown to prevent the differentiation of Th0 cells into proinflammatory Th cells and decrease the secretion of proinflammatory cytokines (IL-2, IFN-γ, TNF-α, IL-17) by Th and Tc cells (168).

Contradictions exist regarding the effects of omega-3 fatty acids on B cell activation, as *in vitro* cultures of B cells isolated from the spleens of mice showed no change in the expression of B cell activation markers, whereas *in vivo* assessment of B cell activation in mice fed a fatty acid-enriched diet showed increased levels of the activation markers CD69, MHC-II, and CD11c (169–171). However, DHA and EPA stimulate IgM production by B cells by increasing the number of antibody-producing cells (172, 173)

An anti-atherosclerotic effect of omega-3 fatty acids has been suggested, as they have been shown to decrease the expression of adhesion molecules by endothelial cells, thereby affecting the migration of leukocytes from the bloodstream into tissues and the subsequent formation of inflammatory foam cells (174).

In a study of 940 children, polyunsaturated fatty acids were shown to reduce the risk of allergic diseases (asthma, rhinitis, and aeroallergen sensitization) (175).

It has been shown that intake of short-chain fatty acids in multiple sclerosis patients or healthy controls promotes the differentiation of Th0 cells into Tregs with increased suppressive capacity (176).

A positive effect of fatty acids on immunity and inflammation has also been demonstrated in breast cancer patients who followed an enriched fish oil diet, including DHA and EPA, resulting in the maintenance of Th cell and serum hsCRP levels, indicating a positive contribution of fatty acids to immune system function and inflammatory response (177). Omega-3 fatty acids have shown a positive effect on attenuating inflammation in patients with chronic kidney disease, as their supplementation resulted in increased production of LTB5 and specialized proresolving lipid mediators, which mediate the resolution of inflammation, and decreased levels of myeloperoxidase, an inflammatory mediator secreted by neutrophils (178).

A significant decrease in the levels of proinflammatory cytokines IL-17A and TNF-α was observed in patients with asthma who received a daily capsule of DHA and EPA, indicating a potent role of fatty acids as a complementary approach in the treatment of this disease (179).

## Future Perspectives

Macro- and micronutrients exert critical and diverse roles in both innate and adaptive immunity by regulating the proliferation, function, and activity of various types of immune cells, as well as their interactions and signal transduction associated with inflammatory responses. Micronutrient deficiencies and malnutrition are common in most countries of the world, highlighting the need for well-organized nutritional care for the general population to prevent disease. Specific guidelines for nutritional care and assessment of patients’ nutritional status are published to guide physicians on the best therapeutic strategies. However, medical nutritional therapy of critically ill patients requires further investigation to describe the relationship between nutrients and patients’ immune physiology (181, 182). Nutritional care of cancer patients undergoing chemotherapy or radiotherapy is a challenge to improve the efficacy and outcomes of cancer treatment and limit disease progression (183). A detailed study of the effect of nutrients on the immune system is an important goal of nutritional immunology (184). Molecular signaling of specific micronutrients is being studied to find drug targets for specific diseases associated with micronutrient deficiency, such as targeting the zinc transporter to treat insulin resistance in type 2 diabetes (185). Studies are being conducted to investigate the effects of specific dietary interventions on human disease progression and chronic inflammation (186). Diets containing nutrients with anti-inflammatory properties have been shown to reduce the risk of depression and attenuate the severity of depression symptoms (187). The study of food consumption in overweight adolescents has revealed the existence of a unique dietary profile in this group associated with obesity (188). The role of nutrition in the management of the severity of COVID-19 and the recovery of surviving patients is currently under investigation; in particular, supplementation of vitamins C, D and zinc to improve patient health, restore immune homeostasis, and reduce the risk of infection for healthy individuals, and probiotics to improve gastrointestinal symptoms in COVID-19 patients (189).

The field is open for further studies that will help to clarify the biological role of food metabolites in the physiology of the organism and especially the immune system, as well as the association of specific dietary nutrients with the pathogenesis of diseases.

## Author Contributions

AM and CT conceived and coordinated the study. ET and AM did the literature search and analysis and wrote the manuscript. AM and CT were responsible for the revision of the manuscript for important intellectual content. All authors contributed to the article and approved the submitted version.

## Funding

ET is a recipient of KARATHEODORIS grant #80672 from the University of Patras. Τhe publication of this article has been financed by the Research Committee of the University of Patras.

## Conflict of Interest

The authors declare that the research was conducted in the absence of any commercial or financial relationships that could be construed as a potential conflict of interest.

## Abbreviations

APC, antigen-presenting cell; APRIL, a proliferation-inducing ligand; ATRA, all-trans-retinoic acid; BAFF, B-cell activating factor; CCR9, chemokine receptor 9; DC, dendritic cell; DHA, dehydroascorbic acid or docosahexaenoic acid; DMB, vitamin D, magnesium and vitamin B12; EPA, eicosapentaenoic acid; GALT, gut-associated lymphoid tissue; LPS, lipopolysaccharide; M cell, microfold cell; NASH, nonalcoholic steatohepatitis; NK cell, natural killer cell; NLRP, Nod-like receptor protein; NO, nitric oxide; PGRP, peptidoglycan recognition receptor protein; ROS, reactive oxygen species; PPs, Peyer’s patches; RAR, retinoic acid receptor; RXR, retinoid X receptor; sCD14, soluble CD14; Tc, T cytotoxic; Th, T helper; TLR, Toll-like receptor; Treg, regulatory T cell; TRX, thioredoxin-like protein;

VDR, vitamin D receptor.
